# Supplementary figures and images for: Contribution of amino acids in the active site of dipeptidyl peptidase 4 to the catalytic action of the enzyme
Source: PLoS One. 2024 Apr 16;19(4):e0289239. doi: 10.1371/journal.pone.0289239 (PMC11020753; doi:10.1371/journal.pone.0289239)

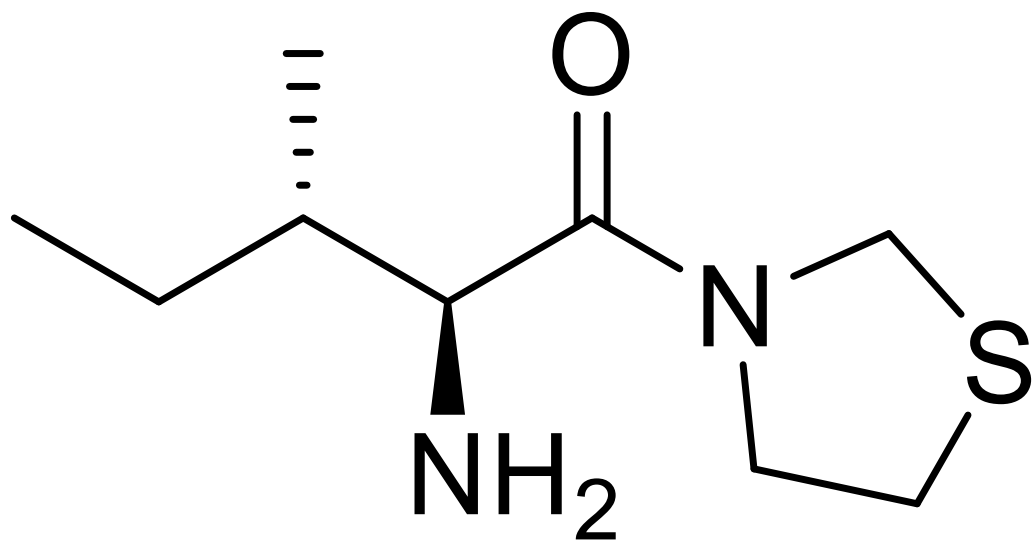

Supplement: S1 Fig — (PDF) [file pone.0289239.s001.pdf]

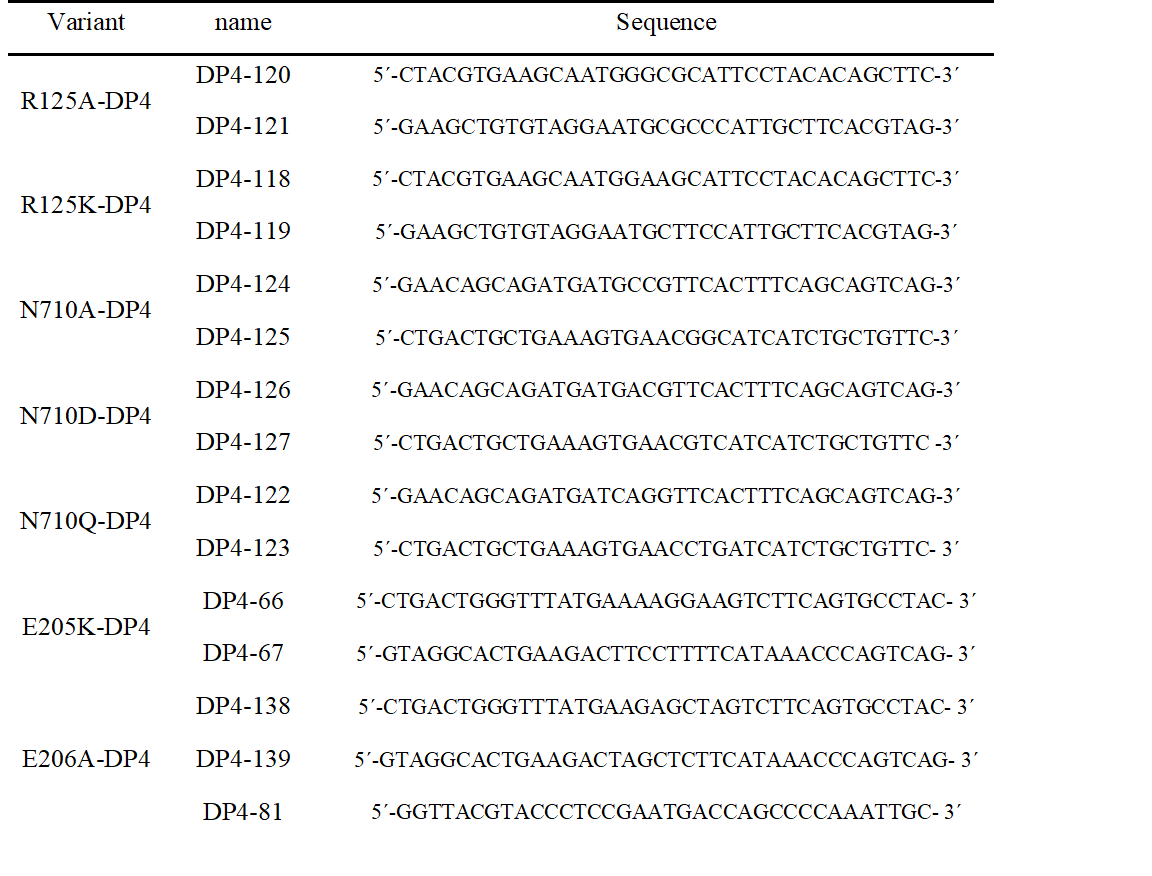

Supplement: S1 Table — All primers to perform side directed mutagenesis were purchased from metabion (Planegg/Steinkirchen, Germany). (TIF) [file pone.0289239.s002.tif]
